# Correlation of the prognostic value of FNDC4 in glioblastoma with macrophage polarization

**DOI:** 10.1186/s12935-022-02688-7

**Published:** 2022-09-02

**Authors:** Hongwu Li, Xiaofei Yan, Shaowu Ou

**Affiliations:** 1 Department of Neurosurgery, First Affiliated Hospital of China Medical University, Shenyang, China; 2 Department of Pathology, The First People’s Hospital of Nantong City (Affiliated Hospital 2 of Nantong University), Nantong, China; 3 Department of Neurosurgery, The First People’s Hospital of Nantong City (Affiliated Hospital 2 of Nantong University), Nantong, China

**Keywords:** Fibronectin type III domain containing 4, Glioblastoma, Prognosis, Macrophage

## Abstract

**Background:**

Glioblastoma is among the most malignant tumors in the central nervous system and characterized by strong invasion and poor prognosis. Fibronectin type III domain-containing 4 (FNDC4) plays various important roles in the human body, including participating in cellular metabolism and inflammatory responses to cardiovascular diseases, influencing immune cells, and exerting anti-inflammatory effects; however, the role of FNDC4 in glioblastoma has not been reported.

**Methods:**

In this study, bioinformatics databases, including TCGA, CGGA, GTEx, and TIMER, were used to analyze the differential expression of *FNDC4* genes and cell survival, in addition to investigating its relationship with immune cell infiltration. Additionally, we overexpressed FNDC4 in glioblastoma cell lines U87 and U251 by lentiviral transfection and detected changes in proliferation, cell cycle progression, and apoptosis. Following collection of monocytes from the peripheral blood of healthy individuals and transformation into M0 macrophages, we performed flow cytometry to detect the polarizing effect of exogenous FNDC4, as well as the effect of FNDC4-overexpressing glioblastoma cells on macrophage polarization in a co-culture system.

**Results:**

We identified that significantly higher *FNDC4* expression in glioblastoma tissue relative to normal brain tissue was associated with worse prognosis. Moreover, we found that FNDC4 overexpression in U87 and U251 cells resulted in increased proliferation and affected the S phase of tumor cells, whereas cell apoptosis remained unchanged. Furthermore, exogenous FNDC4 inhibited the M1 polarization of M0 macrophages without affecting M2 polarization; this was also observed in glioblastoma cells overexpressing FNDC4.

**Conclusions:**

FNDC4 expression is elevated in glioblastoma, closely associated with poor prognosis, and promoted the proliferation of glioblastoma cells, affected the S phase of tumor cells while inhibiting macrophage polarization.

**Supplementary Information:**

The online version contains supplementary material available at 10.1186/s12935-022-02688-7.

## Background

In the central nervous system, glioblastoma is the most common malignant tumor and shows the highest degree of malignancy and mortality [1–3]. The main characteristic of glioblastoma is that it is extremely aggressive [4] and relapses very easily after surgical resection [5]. The prognosis of glioblastoma patients is poor, with a 5-year survival rate of < 10% [6]. Moreover, glioblastoma shows strong heterogeneity [7] accompanied by poor sensitivity to many radiotherapy and chemotherapy drugs [8]. These challenging pathological characteristics result in limited treatment options and highlight the importance of investigating the associated molecular mechanism to identify possible treatment strategies.

Fibronectin type III domain-containing (FNDC) is a highly conserved protein family [9], with five of the eight members found in humans playing important biological roles [10]. FNDC1 and FNDC4 levels are significantly elevated as inflammatory factors and play important roles in inflammatory bowel disease, making them attractive therapeutic targets [11]. In gastric cancer, FNDC1 expression is abnormally elevated and associated with poor prognosis [12], with similar findings reported for breast and pancreatic cancer [13, 14]. Additionally, studies show that FNDC3B plays an important regulatory role in glioblastoma, and that targeted regulation of FNDC3B can effectively inhibit glioblastoma proliferation and invasion [15, 16]. Furthermore, FNDC4 promotes tumor migration and invasion in hepatocellular carcinoma via the phosphoinositide 3-kinase (PI3K)/Akt signaling pathway [17].

Despite the FNDC family showing important regulatory roles in tumors, the role of FNDC4 has not been evaluated in glioblastoma. In this study, we investigated the role(s) of FNDC4 in glioblastoma by combining bioinformatics analysis, functional experiments, and correlation analyses.

## Methods

### FNDC4 expression and survival analysis

FNDC4-expression data were obtained from several public databases, including The Cancer Genome Atlas (TCGA), China Cancer Genome Atlas (CGGA), and the Genotype–Tissue Expression project (GTEx), targeting information concerning grade IV primary glioblastoma.

Expression data in GTEx and TCGA were normalized and merged using the “limma” package in R (https://www.r-project.org/). We then used the “ggstatsplot” package to visualize the differential expression of *FNDC4* in order to evaluate differences between normal brain tissue and glioblastoma tissue according to data from all three databases.

Survival information from patients (survival time and status) was obtained from TCGA and CGGA, respectively. The median *FNDC4*-expression value was segmented into high and low groups, and survival curves were generated using the “survival” and “survminer” packages in R.

### Correlation analysis of FNDC4 expression with the immune microenvironment

We used TCGA data on *FNDC4* expression in glioblastoma to evaluate correlations with immune cell infiltration. The degree of immune invasion in each glioblastoma patient was calculated and scored using the “estimate” package in R. We then used the “ggplot2.” “ggpubr,” and “ggExtra” packages for correlation analyses, visualization of the results, and to generate immune-infiltration scores. Additionally, analyses were performed on *FNDC4*-expression and immune cell-infiltration data from the CIBERSORT database (https://cibersort.stanford.edu/) using the “e1071” and “preprocessCore” R packages.

### Construction and culture of *FNDC4*-overexpressing cells

Glioblastoma cell lines U87 and U251 were purchased from the Cell Bank of the Chinese Academy of Sciences (Shanghai, China). The two cell lines were cultured in a liquid medium comprising 89% Dulbecco’s modified Eagle medium (DMEM; C11995500BT; Gibco, Gaithersburg, MD, USA), 10% fetal bovine serum (FBS; 10,099,141; Gibco), and 1% antibiotics in a 37℃ incubator at 5% CO_2_. We used a lentiviral vector (Genechem Co. Ltd., Shanghai, China) to construct an *FNDC4*-overexpressing cell line. The transfection system comprised 2 mL DMEM, 2 μL polyamine (10 mg/mL), and 50 μL of the lentiviral vector (2 × 10^7^ TU/mL) harboring a puromycin-resistance gene, with 10 μL of an empty lentiviral vector (1 × 10^8^ TU/mL; pLKO.1 puro) used for the control [empty vector (EV) group]. After transfection, purinomycin was used to screen for 1 week, and purinomycin-resistant cells stably overexpressing *FNDC4* were selected. DMEM containing 5 μg/mL purinomycin was used as the culture media in all subsequent experiments.

### Western blotting and enzyme-linked immunosorbent assay (ELISA)

*FNDC4*-overexpressing U87 and U251 cells (FNDC4 OE U87/U251 and EV U87/U251) were collected and digested with radioimmunoprecipitation assay buffer containing protease- or phosphatase-inhibitor cocktails (PC102; Epizyme Biotech, Shanghai, China). Protein concentration was determined using a BCA working solution (ZJ101; Epizyme Biotech) using the ratio of electrophoresis solution to membrane-transfer solution adjusted according to the manufacturer’s instructions (500 ml: 500 ml). We performed sodium dodecyl sulfate polyacrylamide gel electrophoresis (KGP113K; Keygen Biotech Corp., Ltd., Jiangsu, China), followed by transfer of the protein bands to a polyvinylidene fluoride membrane (IPVH00010; Keygen Biotech Corp., Ltd.) and blocking with 5% skim milk. The membranes were incubated with anti-FNDC4 (1:1000; PA5-109,735; Thermo Fisher Scientific, Waltham, MA, USA) and anti-β-actin (1:2000; 66,009–1-Ig; Proteintech, Rosemont, IL, USA) overnight at 4℃, followed by incubation with secondary antibodies (1:10,000) and visualization by chemiluminescence.

We then performed ELISAs to detect exocrine levels of FNDC4 in the cell culture medium. Supernatant from the culture medium was obtained after serum-free starvation of each cell type was mixed with ELISA reagents (MBS7208952; MyBioSource, San Diego, CA, USA). The ELISA microplate was filled with 50 µL of standard solution, a diluted sample (EV: 1:20; FNDC4 OE: 1:200), and 50 µL antibody cocktail. After treatment with 100 µL 3, 3′, 5, 5′-tetramethylbenzidine substrate solution, the experiment was terminated with 100 µL termination solution, followed by determination of absorbance at 450 nm using a microplate reader.

### Cell Counting Kit-8 (CCK-8) and migration assays

EV U87, EV U251, FNDC4 OE U87, and FNDC4 OE U251 cells were seeded with 7.5 × 10^3^ cells per well in 96-well plates for further culturing. Four parallel control groups were set in each group, and they were cultured for 24, 36, 48, 72, and 96 h, respectively. Next, 10 μl CCK-8 reagent was added to each well, and the solutions were allowed to react for 1.5 h; subsequently, the plate was placed in a full-wavelength microplate reader for absorbance measurements at 450 nm.

Transwell chambers (12-well format; Corning) were used to measure cell migration function. The above four kinds of cells were seeded into a chamber with a number of 2 × 10^5^ cells and cultured in a serum-free medium of 200 μl. 800 μl of DMEM containing 10% FBS was added to the lower layer of the chamber and cultured in a constant temperature incubator at 37℃ for 24 h. It was then fixed with methanol and stained with 0.04% trypan blue stain (Solarbio). Five fields were randomly selected under a 200 × microscope for shooting and counting.

### Cell cycle and apoptosis detection

EV U87, EV U251, FNDC4 OE U87, and FNDC4 OE U251 cells were seeded with 2 × 10^5^ cells per well in 96-well plates for further culturing. Then they were cultured for 48 h and digested by trypsin to allow cell precipitation.

We took 1 × 10^5^ of these four cells and used them for cell cycle detection; 1 ml of pre-cooled 70% alcohol was added to fix the cells, and they were treated overnight at 4 ℃. Propidium iodide staining solution containing RNA enzyme A was prepared according to the reagent instructions (Shanghai Beyotime Biotechnology; No. C1052); 0.5 ml of the staining solution was added to each well, and the plate was then placed in a 37 ℃ water bath, and incubated for 30 min away from light. After processing, it was transferred to a BD flow tube until machine testing.

Apoptosis was measured using 1 × 10^5^ cells along with 500 µL binding buffer added to the four cell precipitates, 5 µL Annexin V–fluorescein isothiocyanate (FITC), and 5 µL PI (KGA107; Keygen Biotech Corp., Ltd.). The cells were mixed in a flow tube and incubated at room temperature for 10 min, followed by flow cytometry.

### Isolation and sorting of CD14.^+^ peripheral blood mononuclear cells (PBMCs)

This study was approved by the Ethics Committee of the First Affiliated Hospital of China Medical University (Shenyang, China), and healthy patients provided written informed consent prior to blood collection. Peripheral blood was collected from healthy volunteers and diluted with phosphate-buffered saline (PBS) at a 1:1 (v/v) ratio, followed by the addition of 20 mL Histopaque-1077 (10,771; Sigma-Aldrich, St. Louis, MO, USA) and human lymphocyte-separation solution and another round of dilution [PBS; 1:1 (v/v)]. The upper monocyte layer was obtained after centrifugation 2000 rpm for 25 min at 4 ℃, and erythrocyte lysate was added to the cell suspension to obtain isolated peripheral monocytes after centrifugation. We then added a 2-mL cell suspension (1 × 10^8^ cells/mL) to a flow tube along with 200 μL of reagent (17,858; STEMCELL Technologies, Vancouver, BC, Canada) for incubation at room temperature for 10 min. We then added 200 µL of magnetic beads to the sample for a 3-min incubation and arrangement over a magnetic pole (ThermoFisher), followed by attachment of the cells to the walls of the flow tube. CD14^+^ PBMCs were obtained by removing the flow tube from the magnetic pole and resuspending the cells with PBS.

### Effect of FNDC4 on macrophage polarization

PBMCs were cultured in mixed medium comprising human monocytic colony stimulating factor (100 ng/mL) and 10% FBS–Roswell Park Memorial Institute-1640, and macrophages were obtained after ~ 72 h of culture. Polarized M1 and M2 macrophages were obtained using serum-free medium containing lipopolysaccharide (100 ng/mL; L4391; Sigma-Aldrich) and interleukin (IL)-4 (20 ng/mL; SRP3093; Sigma-Aldrich) after 24 h, respectively. The experiment was divided into three macrophage groups (M0, M0 + LPS, and M0 + IL-4), with human FNDC4 (AG-40B-0213; AdipoGen Life Sciences, San Diego, CA, USA) added to each group to detect its effect on macrophage polarization. After centrifugation, the precipitate was obtained, and 3 mL of flow staining buffer (554,657; BD Biosciences) containing mouse IgG was added for sealing treatment. CD68, CD80, and CD206 are markers of monocyte macrophages, M1 macrophages, and M2 macrophages, respectively. Groups were incubated with FITC-labeled mouse anti-human CD68 (562,117; BD Biosciences), APC-H7-labeled mouse anti-human CD80 (561,134; BD Biosciences), PE-labeled mouse anti-human CD206 (555,954; BD Biosciences), and FITC-labeled mouse IgG (IgG555057; BD Biosciences) and incubated at room temperature in the dark and subsequently detected by flow cytometry.

### Effect of co-culture of overexpressed FNDC4 and macrophages on polarization of macrophages

To evaluate the effect of co-culture on macrophage polarization, isolated monocytes were pretreated with LPS to obtain M1 macrophages, after which EV U87, EV U251, FNDC4 OE U87, and FNDC4 OE U251 cells were seeded into the lower chamber of a 6-well Transwell plate (2 × 10^5^ cells/well), and M1-polarized macrophages were added to the upper layer. Cells were then incubated with the described antibodies (1:100) targeting cell-surface markers and evaluated using flow cytometry.

### Statistical analysis

Statistical analysis was performed using the GraphPad Prism software (v8.0; GraphPad Software, La Jolla, CA, USA). Student’s unpaired *t* test was used for statistical comparisons of the mean differences across data groups, with the data presented as the mean ± standard error, and all experiments performed independently at least three times. Flow cytometry results were analyzed using the ModFit LT software (Verity Software, Topsham, ME, USA) and FlowJo software (FlowJo LLC, Ashland, OR, USA). In addition, all experimental statistical results are presented as mean ± SE. A P-value < 0.05 was considered significant.

## Results

### *FNDC4* expression in glioblastoma and survival analysis

**Fig. 1:**
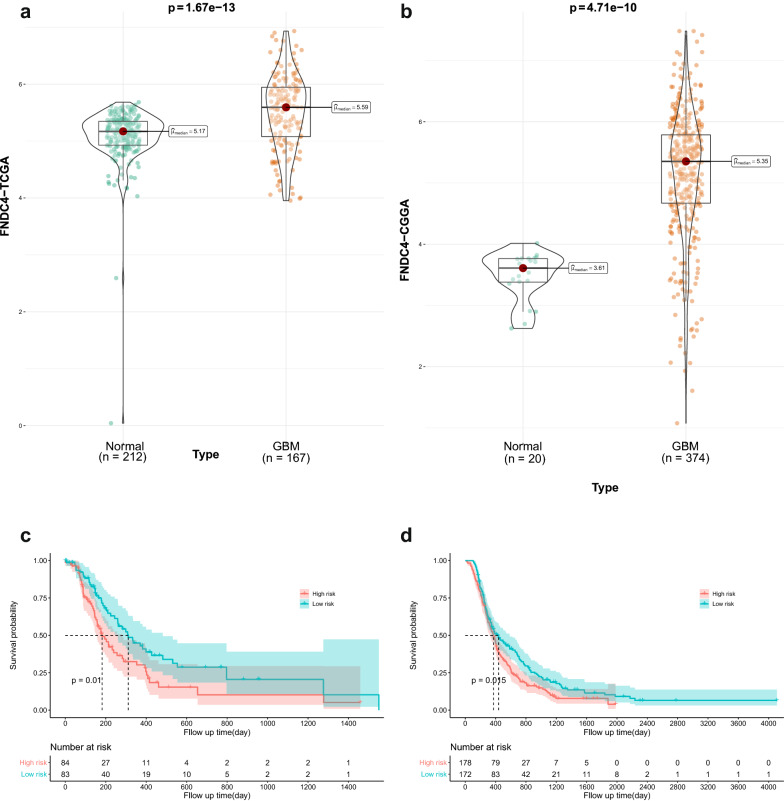
Bioinformatics analysis of FNDC4 in glioblastoma. **a**. Differential expression analysis of FNDC4 from TCGA database. **b**. Differential expression analysis of FNDC4 from CGGA database. **c**. Survival analysis of FNDC4 from TCGA database. **d**. Survival analysis of FNDC4 expression from CGGA database

We extracted *FNDC4*-expression for 212 normal specimens from the GTEx database and combined them with data from 167 glioblastoma patients from the TCGA database. Analyses revealed significant differences in *FNDC4* expression between normal brain tissue and that affected by glioblastoma (P = 1.67e-13) (Fig. 1a); these findings were confirmed using data from the CGGA database (P = 4.71e-10) (Fig. 1b).

We then performed survival analysis using data from TCGA and CGGA. In both databases, high and low expression of *FNDC4* was significantly correlated with glioblastoma patient prognosis (P = 0.01 and P = 0.015, respectively) (Fig. 1c, d).

### Correlation analysis of FNDC4 expression with immune response

**Fig. 2:**
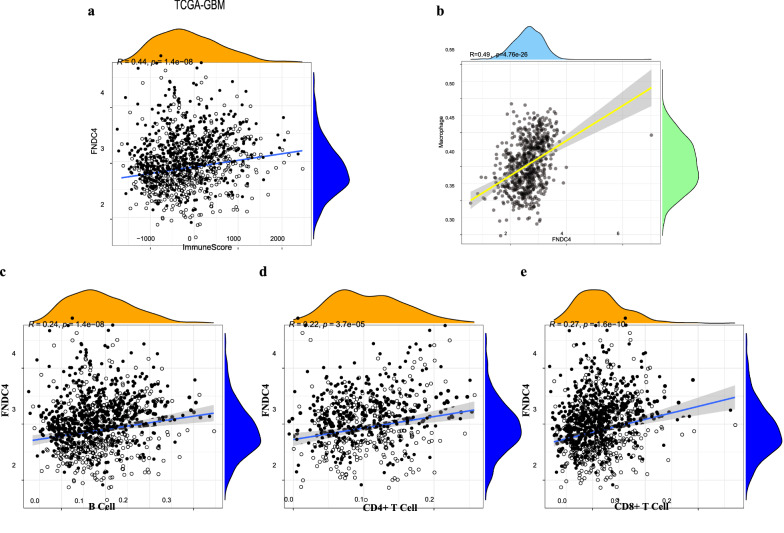
Correlation analysis of FNDC4 expression in immune microenvironment in glioblastoma. **a**. Correlation of FNDC4 expression with immune microenvironment score in glioblastoma. **b**. Correlation analysis between FNDC4 expression and macrophages. **c**. Correlation analysis between FNDC4 expression and B cell. **d**. Correlation analysis between FNDC4 expression and CD4^+^ T cell. **e**. Correlation analysis between FNDC4 expression and CD8^+^ T cell

We found that FNDC4 expression in glioblastoma positively correlated (r^2^ > 0.4) with scores determined for immune cell infiltration (P = 1.4e-08) (Fig. 2a). To clarify the types of infiltrated immune cells, we obtained data from the CIBERSORT database for correlation analysis with FNDC4 expression, which confirmed a significant positive correlation with macrophages (r^2^ = 0.49, Fig. 2b), whereas the correlation with other immune cells was < 0.4 (Fig. 2c, d, e).

### Validation of *FNDC4* expression in glioblastoma cells

**Fig. 3:**
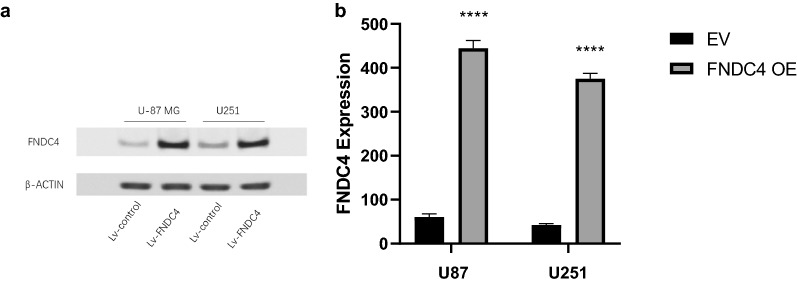
Verification of FNDC4 overexpression efficiency. **a**. FNDC4 overexpression verified by western blotting bands. **b**. Statistical analysis of exocrine FNDC4 overexpression was verified by ELISA. (Data are presented as mean ± SE)

We used U87 and U251 cells to experimentally validate FNDC4 expression using western blot, revealing low FNDC4 levels in both cell lines. Therefore, we overexpressed *FNDC4* in these cell lines via lentiviral vectors and confirmed successful transduction and exocrine secretion (Fig. 3a, b).

### Evaluation of FNDC4 function in glioblastoma

**Fig. 4:**
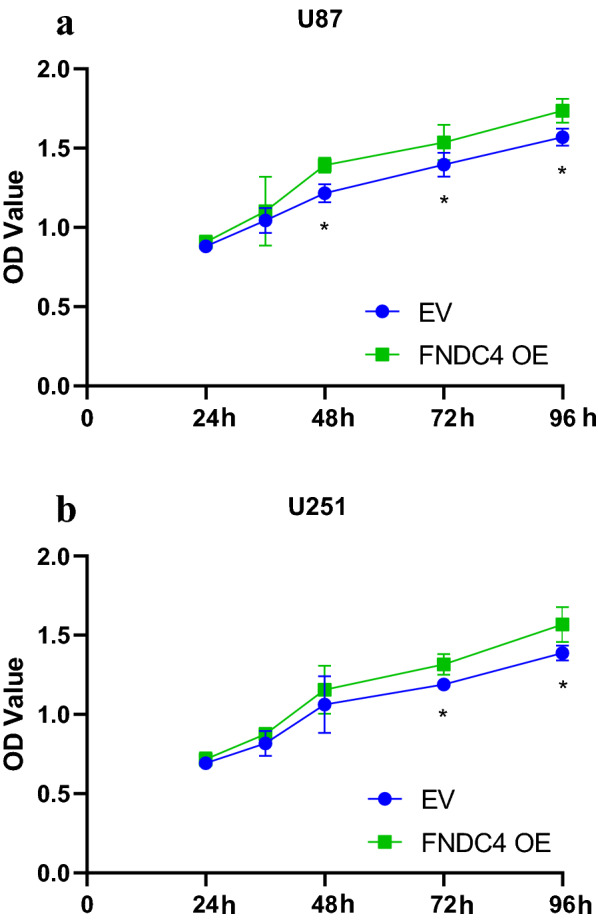
Cell proliferation assay. **a**. Cell proliferation statistics of U87 cell line. **b**. Cell proliferation statistics of U251 cell line

**Fig. 5:**
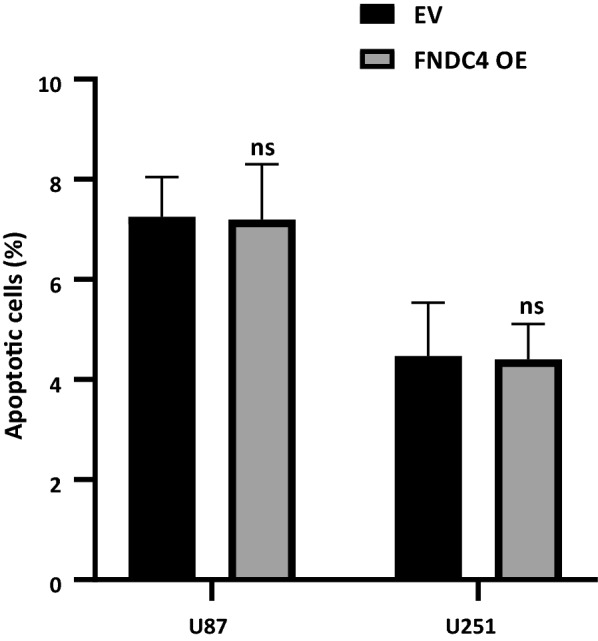
Apoptosis experiment: Statistical analysis of apoptosis experiment data. (Data are presented as mean ± SE)

**Fig. 6:**
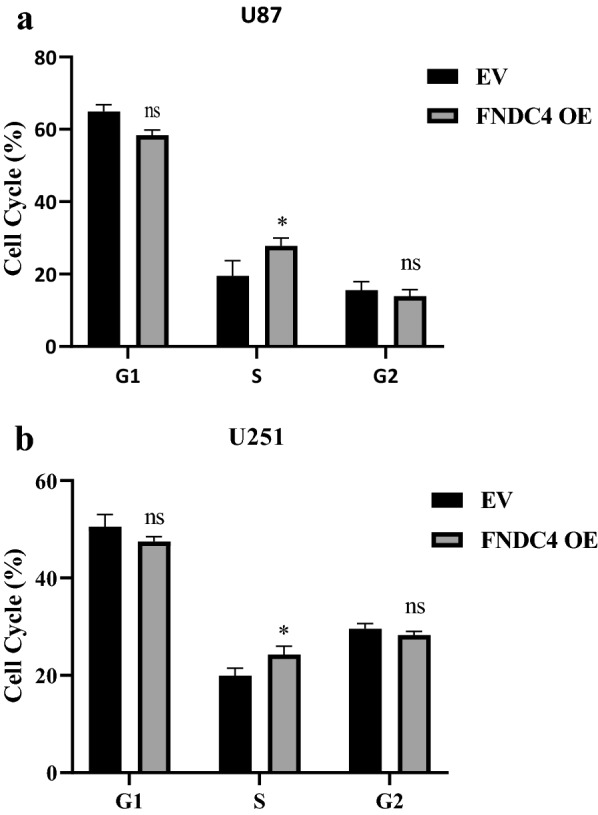
Cell cycle experiment. **a**, **b**. Statistical analysis of cell cycle experiment data. (Data are presented as mean ± SE; “*” represents a p value less than 0.05)

Figure 4 shows that U87 and U251 proliferation was enhanced following *FNDC4* overexpression after 48 h and 72 h, respectively; however, the migration abilities of both cell lines did not change significantly (Additional file 1). Additionally, flow cytometry results showed that *FNDC4* overexpression did not affect glioblastoma cell apoptosis (Fig. 5 and Additional file 2), but affected the S phase of tumor cells (Fig. 6a, b; Additional file 3). Moreover, we found that PI3K/Akt signaling was also unaffected by *FNDC4* overexpression in both cell lines (Additional file 4).

### Exogenous FNDC4 inhibits the M1 polarization of macrophages

**Fig. 7:**
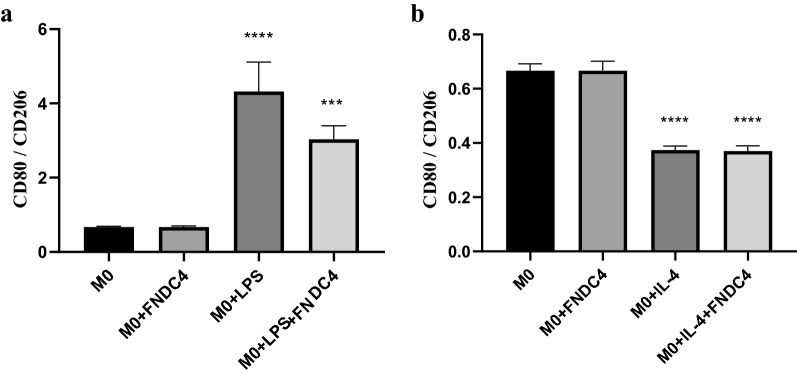
Effect of exogenous FNDC4 on polarization of M0 macrophages. **a**. Analysis of FNDC4 promoting polarization of M0 macrophages towards M1 direction: Compared with M0, the CD80/CD206 ratio of M0 + LPS was significantly increased. Compared with M0 + FNDC4, the CD80/CD206 ratio of M0 + LPS + FNDC4 was also significantly increased. However, compared with M0 + LPS, the CD80/CD206 of M0 + LPS + FNDC4 decreased significantly. **b**. Analysis of FNDC4 promoting polarization of M0 macrophages towards M2 direction: Compared with M0, the CD80/CD206 ratio of M0 + IL-4 was significantly increased. Compared with M0 + FNDC4, the CD80/CD206 ratio of M0 + IL-4 + FNDC4 was also significantly increased. However, compared with M0 + IL-4, the CD80/CD206 of M0 + IL-4 + FNDC4 has barely changed. (Data are presented as mean ± SE; “***” represents p value less than 0.001; “****” represents a p value less than 0.0001)

Following PBMC isolation and induction to M0 macrophages, we evaluated the effect of exogenous FNDC4, revealing almost no change in surface expression of CD80 or CD206 (Additional file 5a, b). Therefore, we used LPS (Additional file 5c) and IL-4 (Additional file 5e) to differentiate M0 macrophages into M1 and M2 subgroups, respectively, followed by exposure to exogenous FNDC4 (Additional file 5d, f). Figure 7a shows that LPS induction increased CD80 expression and decreased CD206 expression in M0 macrophages, resulting in a significantly higher CD80:CD206 ratio than that observed in control M0 macrophages and suggesting LPS-mediated polarization toward M1. Notably, subsequent exposure to exogenous FNDC4 significantly reduced the CD80:CD206 ratio (Fig. 7a), indicating that FNDC4 inhibited M1 polarization. However, Fig. 7b shows that IL-4 induction significantly decreased the CD80:CD206 ratio and promoted M2 polarization, whereas exposure to exogenous FNDC4 did not significantly alter the CD80:CD206 ratio, suggesting no significant effect by FNDC4 on M2 polarization.

### FNDC4 overexpression in glioblastoma inhibits M1 macrophage polarization in the tumor immune microenvironment (TIM)

**Fig. 8:**
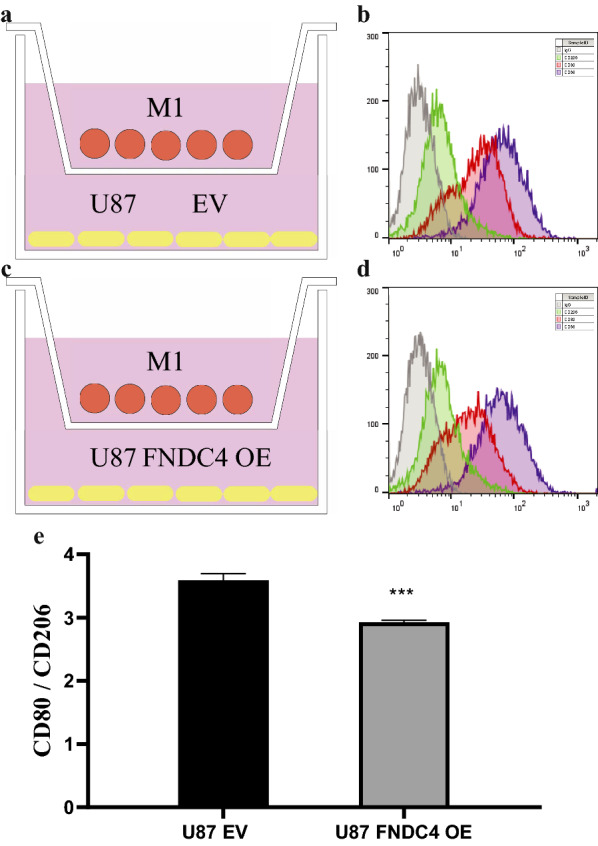
Co-culture experiment of M1 macrophages and U87 cells. **a**, **c**. Schematic diagram of a co-culture of M1 macrophages and U87 cells in the empty vector group and FNDC4-overexpression group. **b**, **d**. Fluorescent antibodies and flow cytometry were used to detect the surface markers of macrophages. (e). Statistical results of the expression of U87 cell-line surface markers. (Data are presented as mean ± SE)

**Fig. 9:**
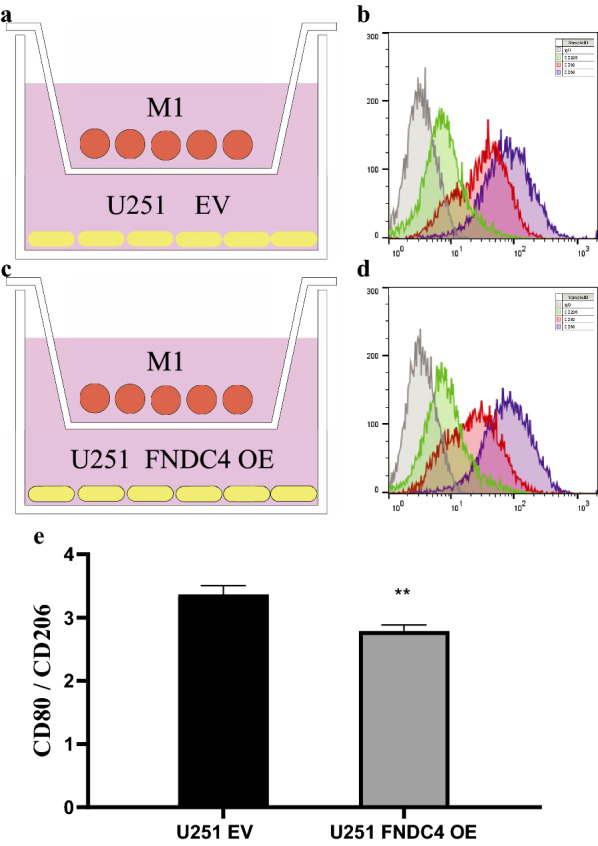
Co-culture experiment of M1 macrophages and U251 cells. **a**, **c**. Schematic diagram of a co-culture of M1 macrophages and U251 cells in the empty vector group and FNDC4-overexpression group. **b**, **d**. Fluorescent antibodies and flow cytometry were used to detect the surface markers of macrophages. **e**. Statistical results of the expression of U251 cell-line surface markers. (Data are presented as mean ± SE.)

We performed Transwell experiments to evaluate the effect of FNDC4 overexpression in U87 and U251 cells on the M1 polarization of M0 macrophages. We found that the CD80:CD206 ratio decreased significantly when co-culturing FNDC4-overexpressing glioblastoma cells with M1 macrophages (Figs. 8e and 9e). These results suggest that FNDC4 overexpression in glioblastoma cells inhibits the M1 polarization of macrophages in the TIM.

## Discussion

FNDC protein family members play an important role in inflammation-related diseases and tumors. For example, FNDC3A can bind FAM46C to inhibit multiple myeloma progression by regulating autophagy [18], and FNDC5 regulates the conversion of white fat to brown fat [19, 20] and improves insulin resistance by regulating the macrophage polarization, thereby reducing the validation response of adipose tissue [21]. Additionally, FNDC5 is a tumor regulator that affects tumor-proliferation and -invasion abilities [22, 23]. Moreover, FNDC4 reportedly plays a role in promoting fat browning, similar to FNDC5 [24], and is an anti-inflammatory factor capable of improving colitis symptoms by specifically binding to macrophages [9, 25, 26]. Based on these findings, we speculated that FNDC4 may play an important regulatory role in tumor status and interactions with the TIM. This represents the first study to explore the potential mechanism and prognostic effect of FNDC4 in glioblastoma. The results showed that FNDC4 promoted tumor proliferation in glioblastoma, affected the S phase of tumor cells and inhibited the polarization of M0 macrophages to the M1 phenotype.

Increasing attention has focused on the role of immune cells in tumors, with the types and subtypes of infiltrating immune cells closely related to tumor occurrence and development [27, 28]. Macrophages are a primary component of the TIM and include in situ macrophages, such as microglia in the brain [26, 29, 30], and monocytes recruited through blood circulation (referred to as tumor-associated macrophages) [31, 32]. In the tumor microenvironment, pathogen-associated molecules, such as LPS, interferon-γ, granulocyte–macrophage colony stimulating factor, and tumor necrosis factor-α, can induce the polarization of M0 macrophages toward an M1 phenotype [33–35]. Upon M1 polarization, macrophages can secrete a variety of cytokines, such as IL-6 and IL-1β, which exert antitumor effects [36]. Additionally, M1 macrophages exhibit antigen-generating capacity and participate in the activation of a T helper cell 1-mediated immune response, thereby producing reactive oxygen species and proinflammatory cytokines to play a defensive role, eliminate tumor cells, and inhibiting tumor progression [37, 38].

In glioblastoma, there exist almost as many small glial cells (i.e., macrophages) as tumor cells [39, 40]. These macrophages secrete important transforming factors, cytokines, and chemokines to activate signaling molecules that induce the proliferation of tumor cells and increase the degree of tumor malignancy [41–43]. In the TIM of glioblastoma, M1-type microglia exert antitumor immune functions by producing proinflammatory cytokines [44, 45] and transcription signals as well as STAT1 [46–48]. However, in the present study, we found that FNDC4 promoted only the proliferation of glioblastoma cells, the potential mechanism associated with which may be related to the observed inhibition of M1 macrophage polarization by exocrine FNDC4. We speculated that in glioblastoma, exocrine FNDC4 plays a vital role in the TIM by suppressing M0 macrophage polarization to M1, thereby inhibiting their antitumor functions and leading to tumor cell proliferation, increased tumor malignancy, and subsequent poor prognosis. At the same time, we also speculated that overexpressed FNDC4 regulates tumor progression and affects tumor proliferation by affecting the S phase of tumor cells.

This study has limitations. Owing to the large number of immune cells present in the TIM, we selected only those identified as correlated with FNDC4 expression for evaluation; therefore, other immune cells need to be investigated. Additionally, the conclusions were obtained from in vitro experiments and, therefore, require validation using in vivo models.

## Conclusions

This study explored the potential mechanism of FNDC4 in glioblastoma through bioinformatics analysis and cell-based experiments. We found that FNDC4 expression in tumor tissues was significantly higher than that in normal brain tissues, and that glioblastoma patients with elevated FNDC4 expression showed poor prognosis. Moreover, we showed that FNDC4 overexpression promoted tumor proliferation and affected the S phase of tumor cells in the absence of significant changes in cell apoptosis progression. Furthermore, the results indicated that exocrine FNDC4 was capable of inhibiting macrophage polarization from an M0 to M1 phenotype, suggesting a potential role in the TIM.

## Supplementary Information

**Additional file 1.** Migration assay. Images of (a, b) U87 cells and (d, e) U251 cells (EV and *FNDC4*-overexpression groups) and (c) U87 and (f) U251 cell migration. Data represent the mean ± standard error.

**Additional file 2.** Apoptosis experiments. Flow cytometric diagram of apoptosis in (a, b) U87 cells and (c, d) U251 cells (EV and *FNDC4*-overexpression groups).

**Additional file 3.** Cell cycle experiments. Flow cytometric diagram of cell cycle progression in (a, b) U87 and (c, d) U251 cells (EV and *FNDC4*-overexpression groups).

**Additional file 4.** PI3K/Akt signaling validation.

**Additional file 5.** Effect of exogenous FNDC4 on macrophage polarization. (a–f) M0 macrophages were divided into groups (M0, M0+LPS, and M0+IL-4) that were treated with exogenous FNDC4 or left untreated. After 24 h, marker expression on the macrophage surface was detected using antibodies and flow cytometry.

**Additional file 6.** Summary of experimental data. CCK-8 and ELISA data.

**Additional file 7.** Western blot results. Uncropped results from Fig. 3a and Supplementary Material 4.

## Data Availability

The data used in this study are all from public databases, including GTEx database (https://gtexportal.org/home/), TCGA database (https://www.cancer.gov/), CGGA database (http://www.cgga.org.cn/) and TIMER database (https://cistrome.shinyapps.io/timer/).

## Abbreviations

FNDC: Fibronectin type III domain-containing
GTEx: Genotype–tissue expression project
TCGA: The Cancer Genome Atlas
OS: Overall survival
CGGA: China Glioma Genome Atlas
CCK-8: Cell Counting Kit-8
EV: Empty vector
DMEM: Dulbecco’s modified Eagle medium
FBS: Fetal bovine serum
PBS: Phosphate-buffered saline
TIM: Tumor immune microenvironment
PBMCs: Peripheral blood mononuclear cells
