# Supplementary figures and images for: Correlation of the prognostic value of FNDC4 in glioblastoma with macrophage polarization
Source: Cancer Cell Int. 2022 Sep 2;22:273. doi: 10.1186/s12935-022-02688-7 (PMC9440505; doi:10.1186/s12935-022-02688-7)

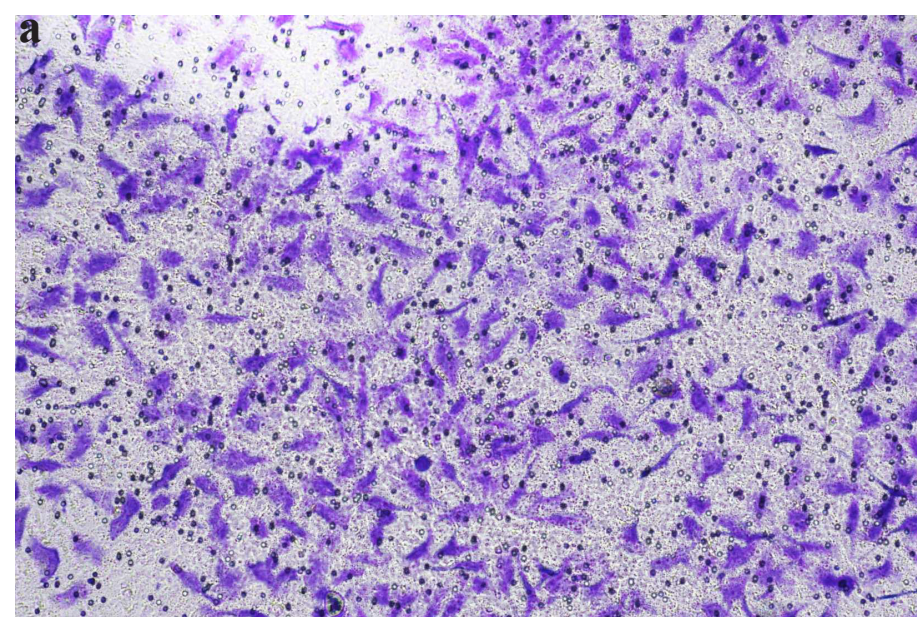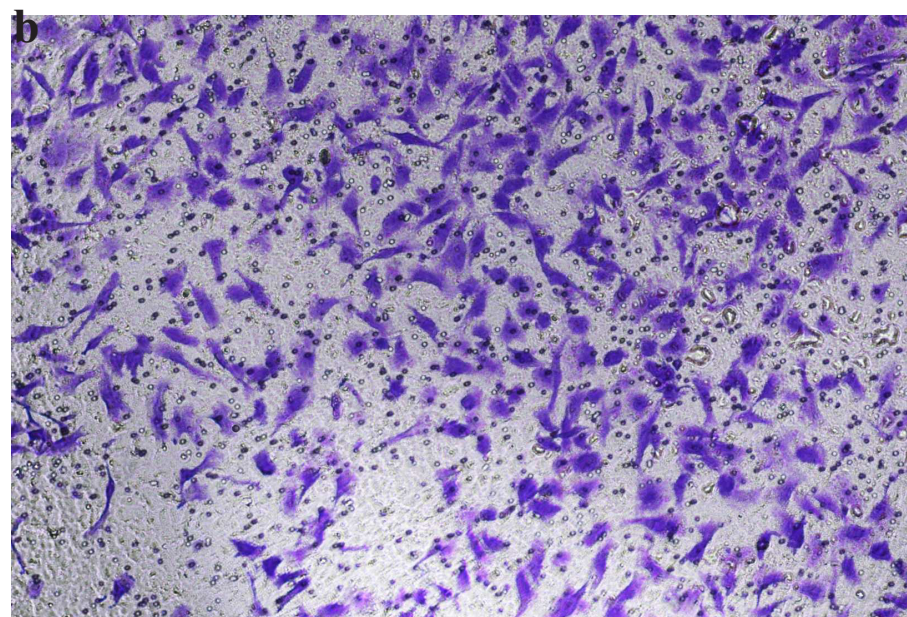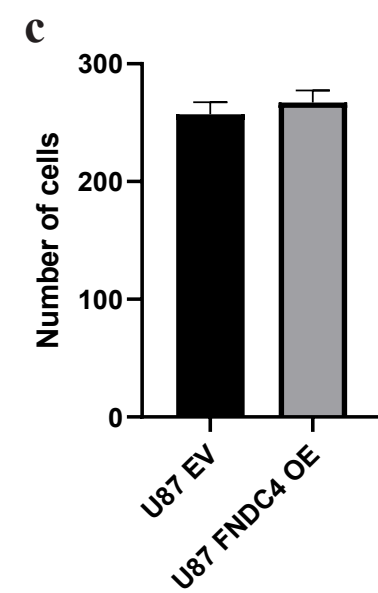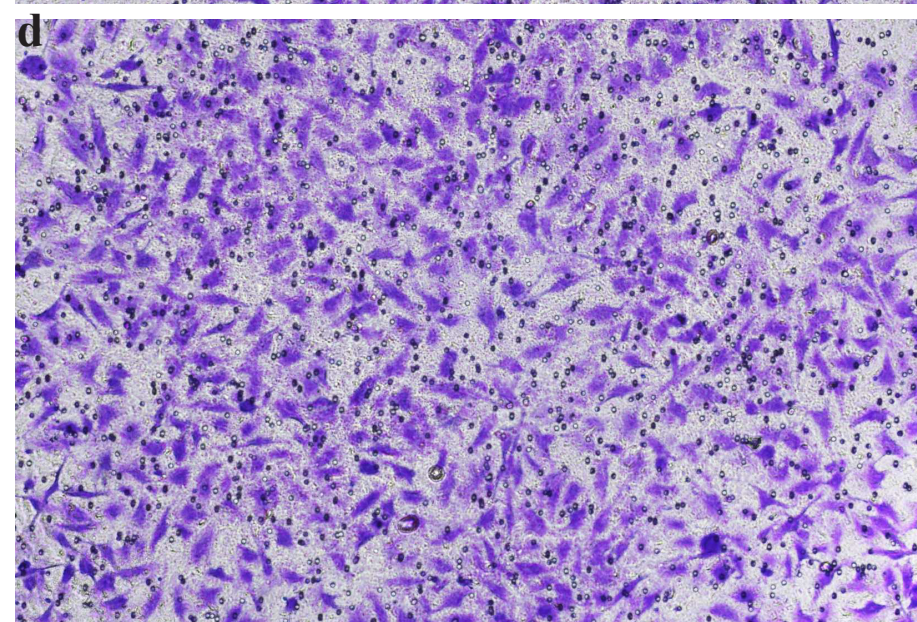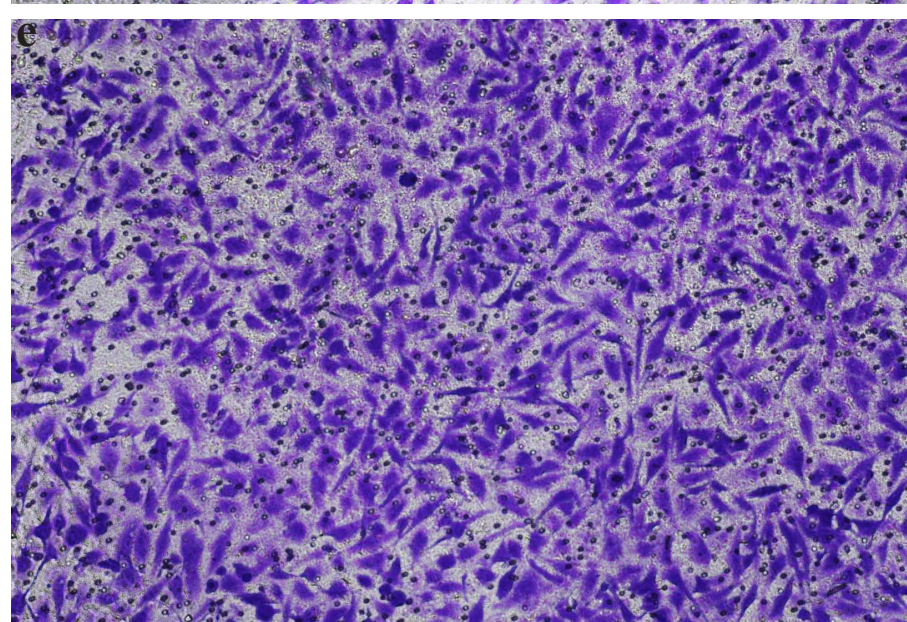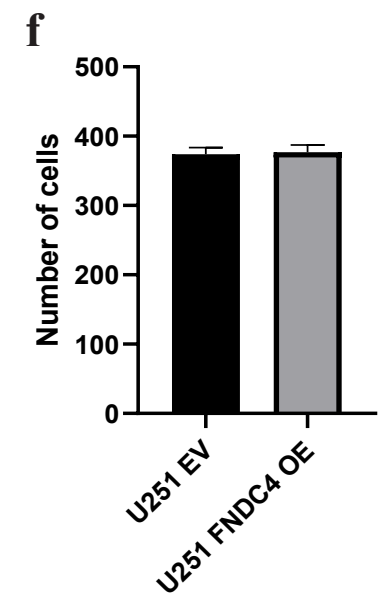

Supplement: Supplementary file 1 — Additional file 1. Migration assay. Images of (a, b) U87 cells and (d, e) U251 cells (EV and FNDC4-overexpression groups) and (c) U87 and (f) U251 cell migration. Data represent the mean ± standard error. [file 12935_2022_2688_MOESM1_ESM.pdf]

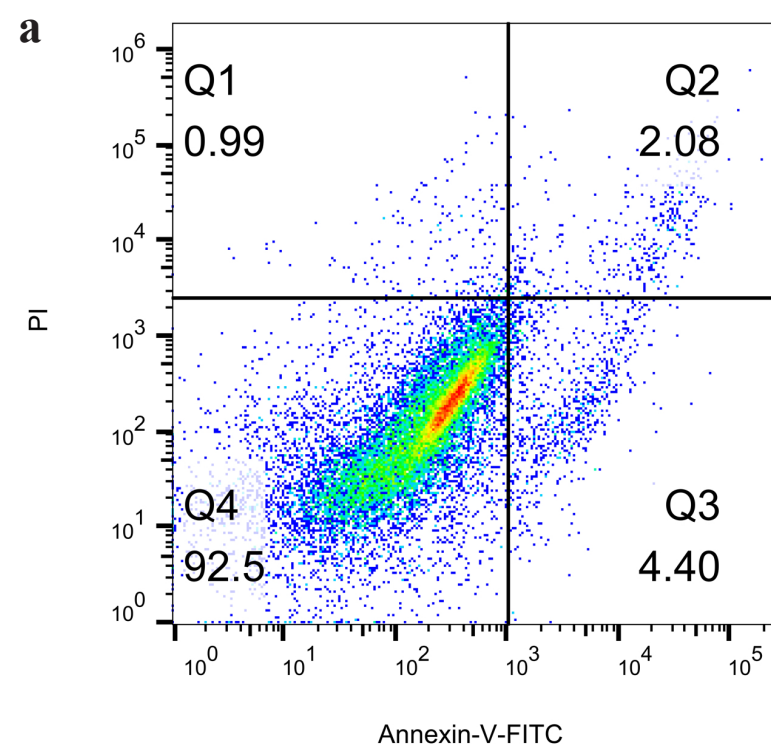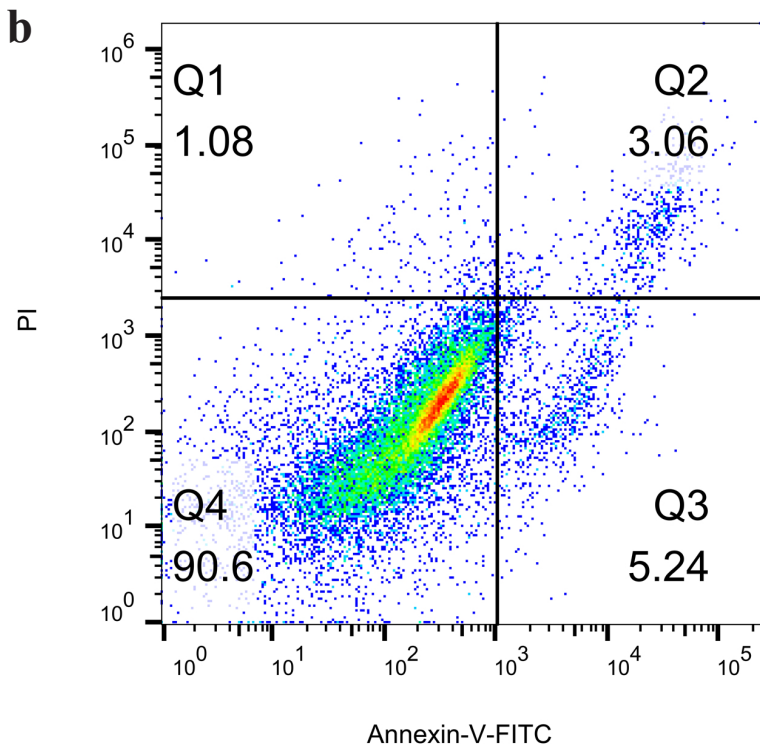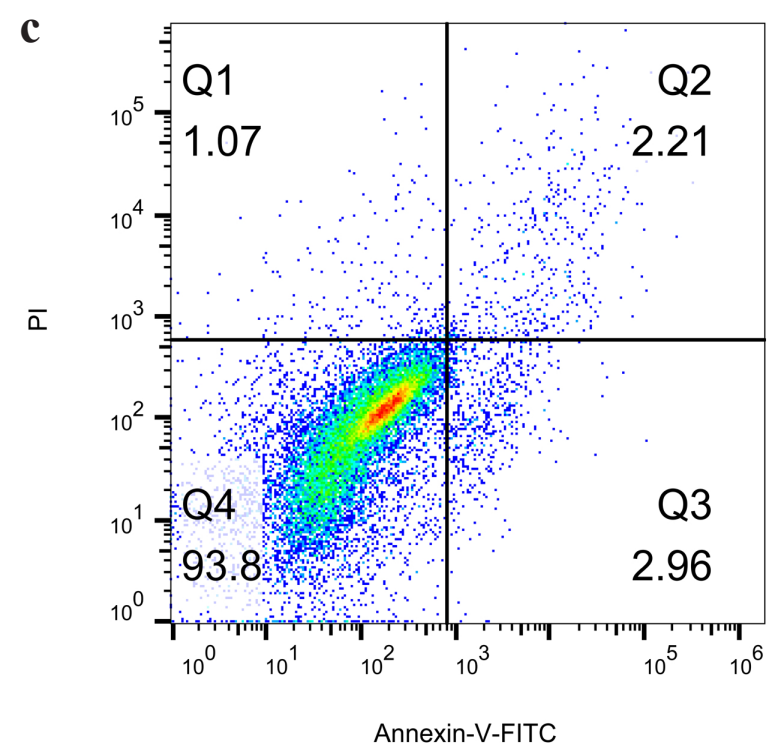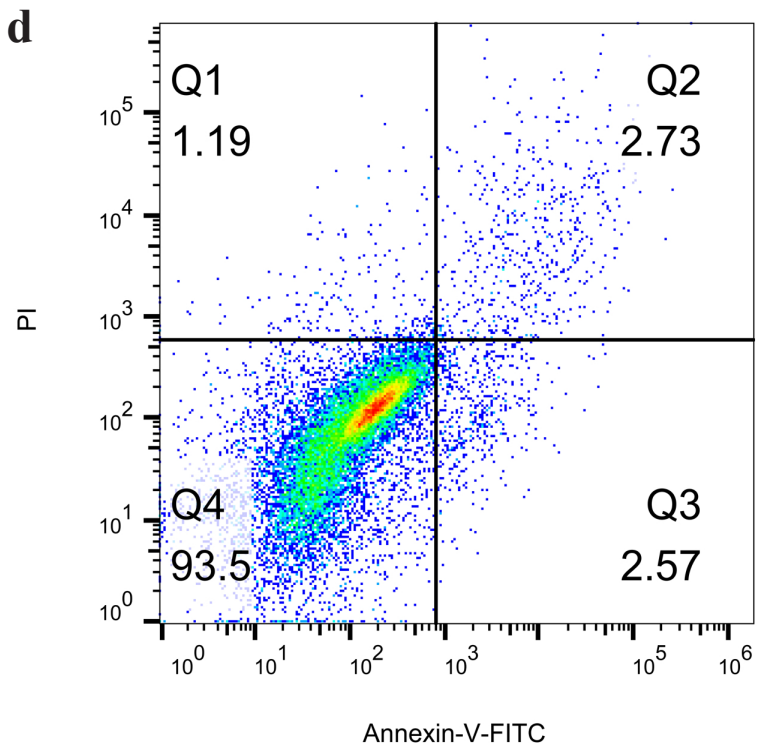

Supplement: Supplementary file 2 — Additional file 2. Apoptosis experiments. Flow cytometric diagram of apoptosis in (a, b) U87 cells and (c, d) U251 cells (EV and FNDC4-overexpression groups). [file 12935_2022_2688_MOESM2_ESM.pdf]

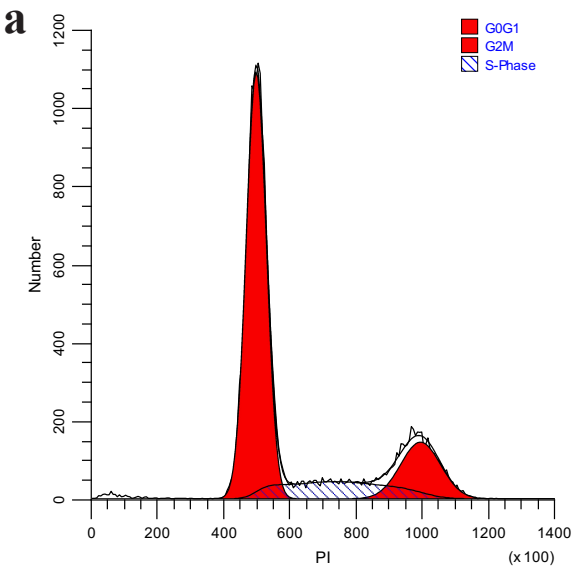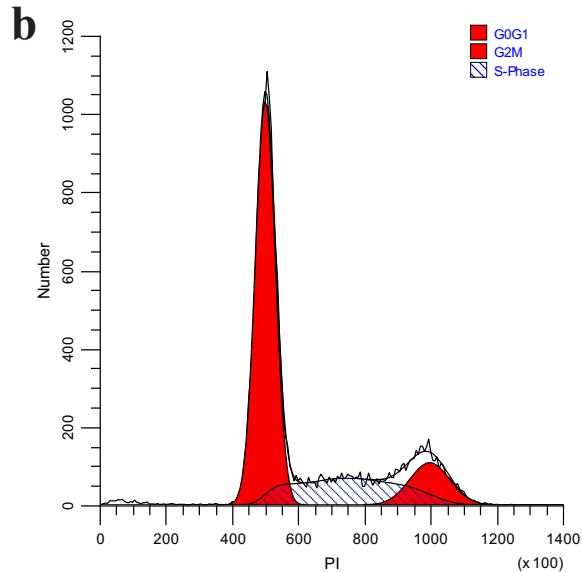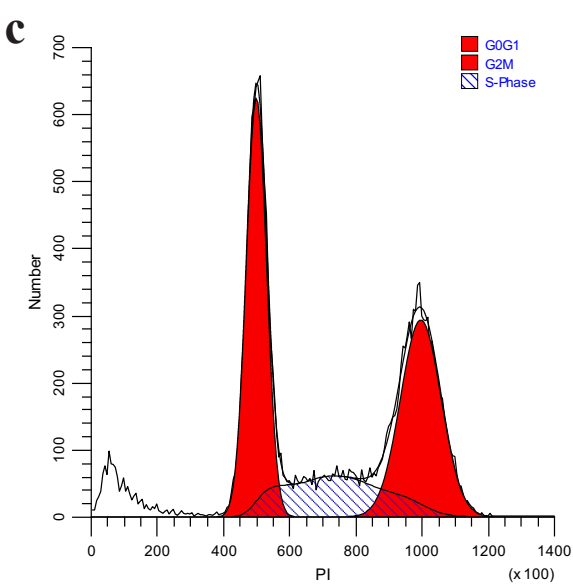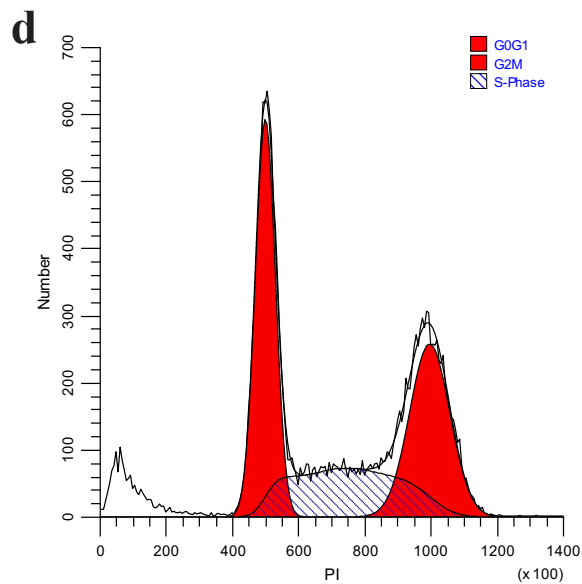

Supplement: Supplementary file 3 — Additional file 3. Cell cycle experiments. Flow cytometric diagram of cell cycle progression in (a, b) U87 and (c, d) U251 cells (EV and FNDC4-overexpression groups). [file 12935_2022_2688_MOESM3_ESM.pdf]

**U87**

**U251**

**pAkt**

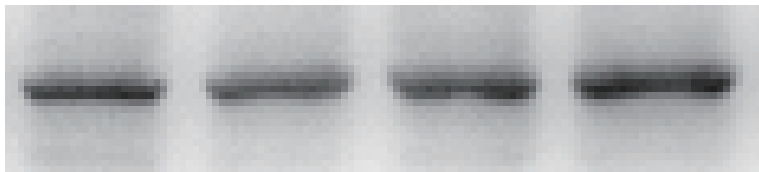

**GAPDH**

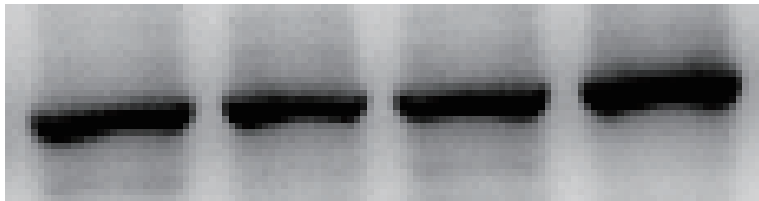

**EV**

**OE**

**EV**

**OE**

Supplement: Supplementary file 4 — Additional file 4. PI3K/Akt signaling validation. [file 12935_2022_2688_MOESM4_ESM.pdf]

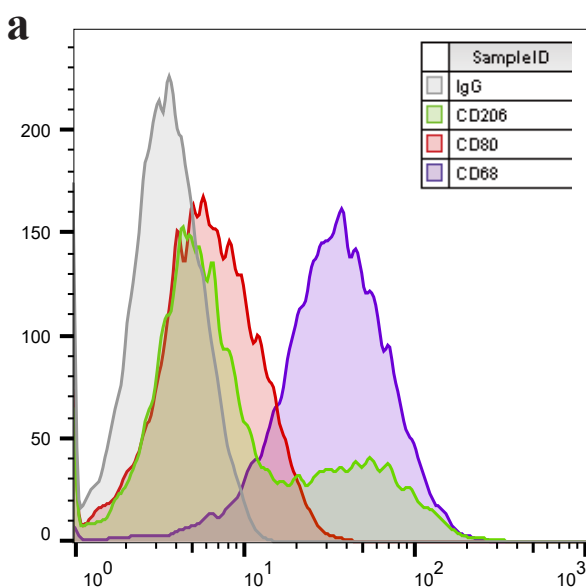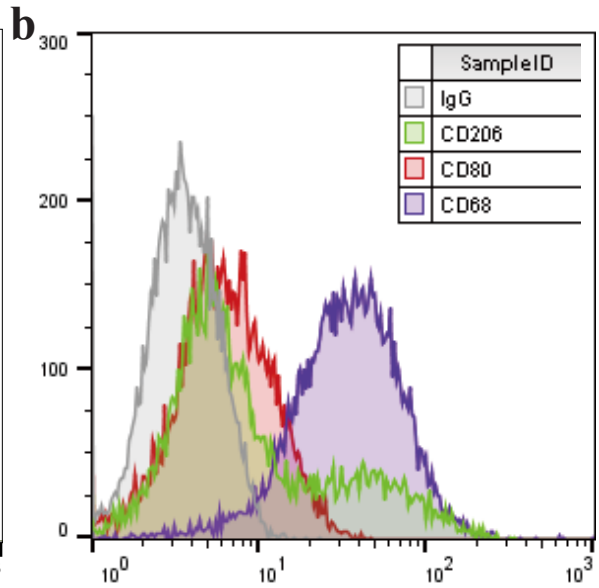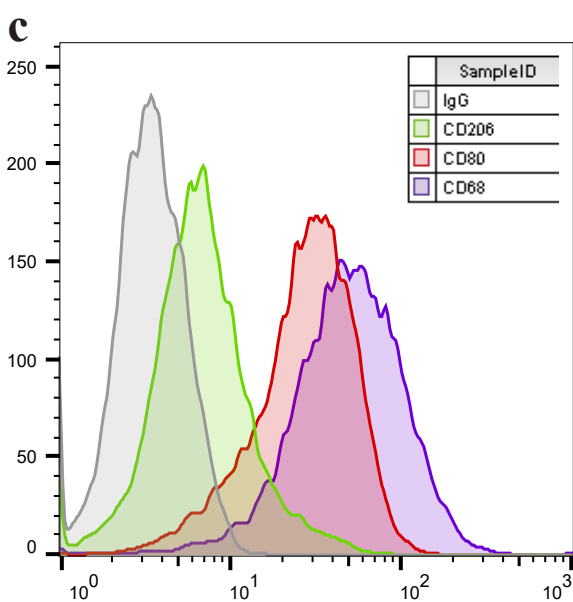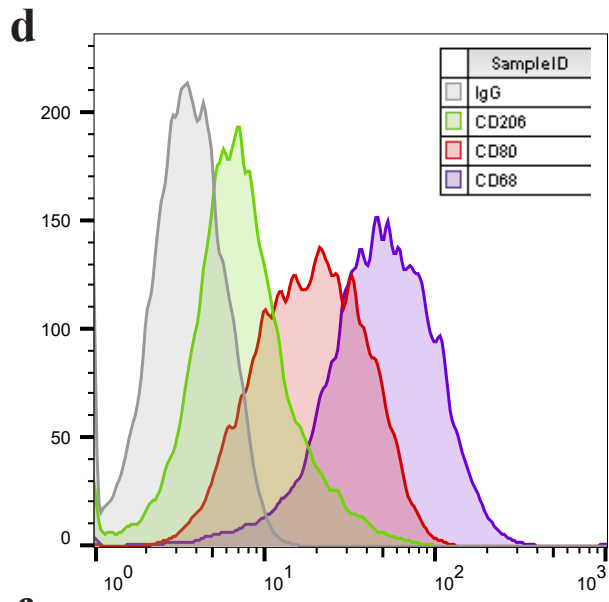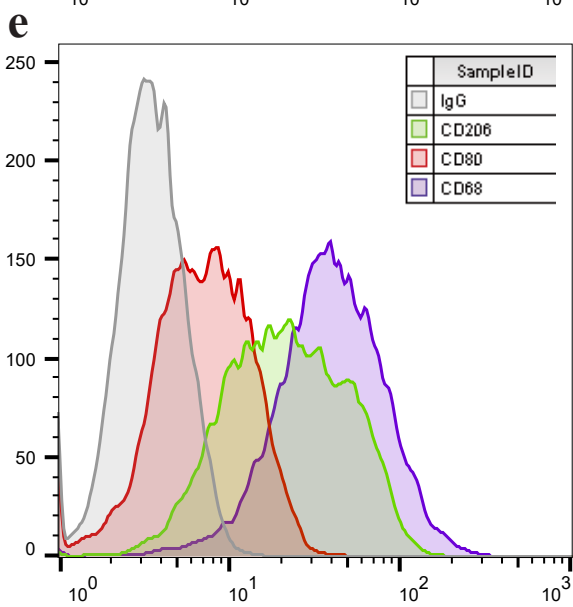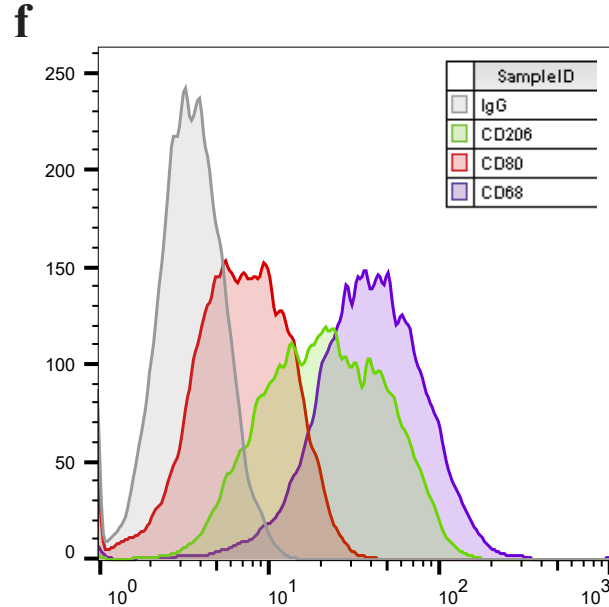

Supplement: Supplementary file 5 — Additional file 5. Effect of exogenous FNDC4 on macrophage polarization. (a–f) M0 macrophages were divided into groups (M0, M0+LPS, and M0+IL-4) that were treated with exogenous FNDC4 or left untreated. After 24 h, marker expression on the macrophage surface was detected using antibodies and flow cytometry. [file 12935_2022_2688_MOESM5_ESM.pdf]

**a**

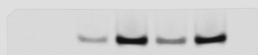

**b**

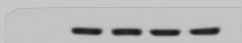

**c**

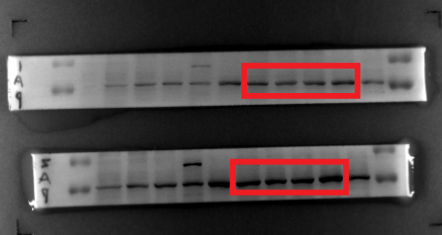

Supplement: Supplementary file 7 — Additional file 7. Western blot results. Uncropped results from Fig. 3a and Supplementary Material 4. [file 12935_2022_2688_MOESM7_ESM.pdf]
